# Expression and function of myelin expression factor 2 in hepatocellular carcinoma

**DOI:** 10.1186/s12876-023-02644-3

**Published:** 2023-01-19

**Authors:** Peng Zhang, Jiang-Hua Zhao, Lin Chen, Zhao-Lian Bian, Lin-Ling Ju, Hui-Xuan Wang, Wei-Hua Cai

**Affiliations:** 1 Medical School of Nantong University, Nantong Third People’s Hospital, Nantong, 226000 Jiangsu People’s Republic of China; 2 Nantong Institute of Liver Diseases, Nantong Third People’s Hospital, Nantong University, Nantong, 226000 Jiangsu People’s Republic of China; 3 Nantong Institute of Liver Disease, Department of Hepatobiliary Surgery, Nantong Third People’s Hospital, Nantong University, Nantong, 226000 Jiangsu People’s Republic of China

**Keywords:** Hepatocellular carcinoma, MYEF2, Prognosis, Migration, Invasion

## Abstract

**Introduction:**

Hepatocellular carcinoma (HCC) is one of the most common malignant tumours in the world and has a high mortality rate. However, the pathogenesis of HCC remains unclear. This study aimed to investigate the potential biomarkers of HCC.

**Methods:**

ONCOMINE, HCCDB and THE HUMAN PROTEIN ATLAS were used to identify myelin expression factor 2 (MYEF2) as a potential biomarker for HCC. The Cancer Genome Atlas database was used to further validate and analyse the value of MYEF2. Kaplan–Meier Plotter was used for the prognostic analysis. The COX regression model and Kaplan–Meier method were used to investigate the clinical value of MYEF2 in the prognosis of HCC by reviewing the survival status of patients. Fluorescent quantitative polymerase chain reaction (qPCR) and immunohistochemistry were used to detect the expressions of the MYEF2 mRNA and protein in HCC tissues and cell lines. qPCR and Western blotting were used to validate the efficiency of MYEF2 knockout and overexpression in HCC cells. The invasion and migration abilities regulated by MYEF2 were detected by performing transwell and wound healing assays.

**Results:**

MYEF2 is significantly upregulated in HCC and is mainly located in the nucleus of HCC cells. MYEF2 expression is significantly associated with the tumour stage, histological grade and TNM stage. High MYEF2 expression is an independent prognostic factor for patients with HCC. Functionally, elevated MYEF2 facilitated cell migration and invasion in vitro. In contrast, decreased MYEF2 inhibited cell migration and invasion.

**Conclusions:**

MYEF2 may be a novel biomarker with potential diagnosis and prognosis values and as a potential therapeutic target for HCC.

**Supplementary Information:**

The online version contains supplementary material available at 10.1186/s12876-023-02644-3.

## Introduction

Liver cancer is one of the most common malignant tumours in the world. According to global cancer statistics in 2020, the incidence of liver cancer ranked seventh in the world, and the mortality rate ranked third. The annual number of new cases of liver cancer is approximately 900,000, and the annual number of new deaths is approximately 830,000 [1, 2]. HCC accounts for more than three-quarters of liver cancer cases [3]. The main risk factors for HCC include hepatitis virus infection, aflatoxin exposure, alcohol consumption, obesity, and smoking [4, 5]. The occurrence and development of HCC is a complex process, and its potential mechanism remains unclear. At present, surgical resection and liver transplantation are still the main treatments for HCC. However, HCC is often diagnosed in the middle and late stages of the disease. Most patients have lost the opportunity for surgery and have a poor prognosis [6–8]. New biomarkers are urgently needed to improve diagnostic accuracy and better predict the prognosis [9]. Therefore, a search for molecular targets related to the occurrence and development of hepatocellular carcinoma has important clinical significance [10].

In this study, we aimed to identify and study candidate biomarkers for HCC. First, we used existing public databases (ONCOMINE, HCCDB, THE HUMAN PROTEIN ATLAS and Kaplan–Meier Plotter) to screen a potential biomarker, MYEF2. At present, no report has been documented the potential value of MYEF2 in determining the diagnosis and prognosis of HCC. Subsequently, the value of MYEF2 was analysed by data mining based on The Cancer Genome Atlas database. We used liver cancer tissues from local patients for experimental validation of the reliability of selected biomarkers based on regional and ethnic differences. In addition, we preliminarily analysed the function of MYEF2 by performing in vitro experiments. Our current study showed that MYEF2 might be a novel tumour marker for HCC.

## Materials and methods

### ONCOMINE

The ONCOMINE database (https://www.oncomine.org/resource/login.html) is currently the world's largest cancer gene chip database and integrated data mining platform with the most complete cancer mutation spectrum, gene expression data and related clinical information [11]. In this study, we searched for differentially expressed genes in HCC. We set the *P* value to less than 0.05, and the fold change to greater than 2. Combined with the existing literature retrieval platform, we searched for liver cancer biomarkers that have not been studied and reported.

### HCCDB database

HCCDB (http://lifeome.net/database/hccdb/home.html) is a web-based database of the HCC expression atlas that includes 15 public HCC gene expression datasets and a total of 3917 samples [12, 13]. We used HCCDB to further study MYEF2 expression in HCC.

### THE HUMAN PROTEIN ATLAS

THE HUMAN PROTEIN ATLAS (https://www.proteinatlas.org/) is a Swedish program launched in 2003 that provides the distribution of human proteins in tissues and cells. The distribution and expression of each protein in normal human tissues, tumour tissues, cell lines and blood were examined using immunohistochemistry [14, 15]. In the present study, we used THE HUMAN PROTEIN ATLAS database for protein expression profiling.

### The cancer genome atlas database

The Cancer Genome Atlas database (https://tcga-data.nci.nih.gov/tcga/) is the largest part of the International Cancer Genome Consortium (ICGC) research plan, which is mainly designed to obtain a comprehensive, multidimensional map for a variety of cancer genomes [16]. In this study, we obtained RNA-seq data and clinical data on MYEF2. Ethical approval was not needed because all data were publicly available.

### Kaplan–Meier plotter database

The Kaplan–Meier plotter database (http://kmplot.com/analysis/) included studies on 54,675 genes and 18,674 cancer samples and evaluates the effects of 54,000 genes on survival rates in patients with 21 cancers [17]. In the present study, we analysed the correlation between the prognostic value of MYEF2 in determining the survival of patients with HCC, along with other factors.

### Patients and tumour tissues

From January 2008 to December 2016, 49 pairs of HCC tissues and corresponding normal tissues were selected from patients with HCC who underwent surgical treatment at Nantong Third Hospital Affiliated with Nantong University. The distance between the tumour tissue and normal tissue was greater than 2.5 cm. After in vitro experiments, the tumour tissues were immediately frozen at − 80 °C until use. In addition, 142 pairs of tissues were fixed with 10% neutral formalin buffer for 24 h, embedded in paraffin and prepared as paraffin sections for preservation. The diagnosis of all patients with HCC was confirmed according to the HCC guidelines for diagnosis and treatment of the European Association [18]. This study was approved by the Ethics Committee of Nantong Third Affiliated Hospital of Nantong University. All patients signed informed consent forms.

### Cell lines and culture

Normal human liver cells HL-7702 (L-02) and Hep 3B2.1-7, SK-HEP-1, HuH-7, and PLC/PRF/5 liver cancer cells were provided by the cell bank of the Chinese Academy of Sciences (Shanghai, China) and cultured at the Nantong Hepatology Research Institute. Hep 3B2.1-7, SK-HEP-1 and PLC/PRF/5 cells were cultured in MEM (NaHCO3 and sodium pyruvate were added) containing 10% foetal bovine serum (FBS; Cell Sciences, Canton, MA). L-02 cells were cultured in RPMI-1640 (Gibco, Thermo Fisher Scientific, Waltham, MA) containing 10% FBS. HuH-7 cells were cultured in Dulbecco’s modified Eagle’s medium (Gibco, Thermo Fisher Scientific) containing 10% FBS. All cells were cultured in a humidified incubator with 5% CO_2_ at 37 °C.

### RNA isolation and fluorescent quantitative polymerase chain reaction (qPCR)

The experimental scheme was the same as the previous research of our research group [19–21]. After total RNA was isolated from tissues and cells by RNAiso Plus (Takara, Beijing, China), the concentration and purity of the isolated RNA were detected by UV-1800 spectrophotometer (Shimadzu Corporation, Kyoto, Japan). Next, the isolated RNA was converted into complementary DNA (cDNA) using a PrimeScript RT Reagent kit (Perfect Real Time; Takara Biotechnology Co., Ltd.). Reaction conditions: 37 °C, 15 min; 85 °C, 5 s, cool to 4 °C. qPCR was performed using a SYBR-Green PCR Master Mix (Vazyme Biotech Co., Ltd., Nanjing, China). qPCR was initially performed at 95 °C for 5 min, and then samples were subjected to 40 cycles of amplification at 95 °C for 10 s and at 60 °C for 30 s. β-actin was used as an internal control gene. The fold amplification for each gene was calculated using the 2^−ΔΔCq^ method. The primer sequences were as follows: MYEF2 forward 5′-GATTTTTATCGGGTCCAATGGG-3′, reverse 5′-ACAGCCTTTTGA CTTTCCATTC-3′; β-actin forward 5′-GGACTTCCGAGCAAGAGATGG-3′, reverse 5′-AGGAAGGAAGGCTGGAAGA-3′.

### Western blotting

Liver cells were lysed with RIPA lysis buffer containing a phosphatase inhibitor cocktail (Beyotime Institute of Biotechnology, Shanghai, China). Then, the samples were centrifuged at 10,000×*g* for 10 min at 4 °C, and the supernatant was collected. A BCA protein assay kit (Beyotime Institute of Biotechnology, Shanghai, China) was used to measure the protein concentration. Equal amounts of protein were electrophoretically separated on 10% SDS–PAGE gels and transferred to nitrocellulose membranes at 100 V for 90 min. β-Actin (cat. no. ab8227; 1:2000; Abcam) was used as an internal control protein, and a specific primary antibody (cat. no. 16051-1-AP; 1:5000; Proteintech) was used to analyse the expression of MYEF2. After an overnight incubation at 4 °C, the membranes were incubated with a horseradish peroxidase-conjugated goat anti-rabbit IgG secondary antibody (cat. no. KC-RB-035; 1:3000; Kangchen Biotech Co., Ltd., Shanghai, China) at room temperature for 1 h. The membranes were washed three times with Tris-buffered saline containing Tween-20 and visualized using an enhanced chemiluminescence system (Thermo Fisher Scientific, Inc.). ImageJ software 1.46 (National Institutes of Health, Bethesda, MD, USA) was used to analyse the grey value of protein bands. Due to the impact of polyclonal antibodies, we cannot provide cleaner, smoother backgrounds and bands. To make the experimental results clearer and more aesthetically pleasing, our experimental results were cut and highly exposed by using the drawing software. We provided original images of full-length blots, which is included in the Additional file 1.

### Immunohistochemistry

The paraffin sections were dewaxed with a series of xylene solutions and gradient of ethanol concentrations. The dewaxed tissue slices were dried in a microwave in citrate buffer (pH 6.0) for antigen retrieval. Then, we incubated the sections with 3% H_2_O_2_ for 15 min to block endogenous peroxidase activity. Slices were incubated with a rabbit anti-human MYEF2 antibody (cat. no. 16051-1-AP; 1:200; Proteintech) overnight at 4 °C. The next day, a horseradish peroxidase-conjugated anti-mouse/rabbit secondary antibody (cat. no. D-3004; Shanghai Changdao Biotechnology Co., Ltd., Shanghai, China) was incubated with the sections for 30 min at room temperature. After washes with PBS, 3,3′-diaminobenzidine (DAB, Maixin-Bio, Guangzhou, China) was used for staining at room temperature for 18 s, followed by haematoxylin staining. Finally, two researchers evaluated all stained sections in a blinded manner. The staining intensity was graded from 0 to 3:0 (no staining), 1 (weak staining), 2 (medium staining) and 3 (strong staining).

### SiRNA transfection

For MYEF2 knockdown, Genepharma (Suzhou City, Jiangsu Province, China) designed and constructed two siRNAs specifically targeting MYEF2 and a negative control (Si-NC). According to the instructions, siRNA (10 μM) was transfected into SK-HEP-1 and Hep 3B2.1-7 cells with Lipofectamine™ 2000 (Thermo Fisher Scientific). The transfection efficiency was verified by performing PCR and western blotting.

### Plasmid transfection

For MYEF2 overexpression, Genepharma designed and constructed a plasmid (pc-DNA3.1-MYEF2) for the specific overexpression of MYEF2 and an empty plasmid (pc-DNA3.1-NC). According to the instructions, the plasmid (2.5 μM) was transfected into PLC/PRF/5 cells using Lipofectamine™ 3000 (Thermo Fisher Scientific). The transfection efficiency was verified by performing qPCR and western blotting.

### Wound healing assay

Cell migration was detected using wound healing assay. In brief, we seeded the transfected cells into 6-well plates. The cells were then scratched on the monolayer using a 200 μl suction head. After removing dead cells with PBS, the cells were cultured in serum-free medium. We photographed and recorded the scratch width at the specified time. GraphPad Prism 9.0 software was used to visualize the results.

### Transwell assay

Cell migration and invasion were detected using Transwell chambers coated without or with Matrigel (BD Biosciences, San Jose, CA, USA). Matrigel was coated on the basement membrane of the Transwell chamber in the invasion assay. A serum-free cell suspension containing 2 × 105 hepatoma cells was inoculated into the upper chamber (8 μm, Millipore). Medium containing 20% FBS was added to the bottom chamber as a chemoattractant. After a certain period of culture at 37 °C with 5% CO_2_ and saturated humidity, the cells remaining in the upper chamber were removed, and the cells that migrated through the membrane were stained with crystal violet (Beyotime) after fixation with methanol. The number of cells that migrated or invaded was calculated from five randomly selected fields using an optical microscope (Olympus, Japan). Cell migration time: SK-HEP-1 cells, 20 h; PLC/PRF/5 cells, 14 d. Cell invasion time: SK-HEP-1 cells, 24 h; PLC/PRF/5 cells, 15 d.

### Statistical analysis

In this study, SPSS 26.0 and GraphPad Prism 9.0 software were used for data analysis and visualization. In this study, the enumeration data were analysed using the chi-square test, the measurement data were analysed using the independent sample T test, and the nonparametric rank sum test was used for data with nonnormal distributions. The survival analysis was performed using Kaplan–Meier method and log rank tests. Univariate and multivariate analyses were performed using the Cox regression model. *P* < 0.05 was considered significant.

## Results

### Preliminary screening of liver cancer biomarkers in public databases

**Fig. 1:**
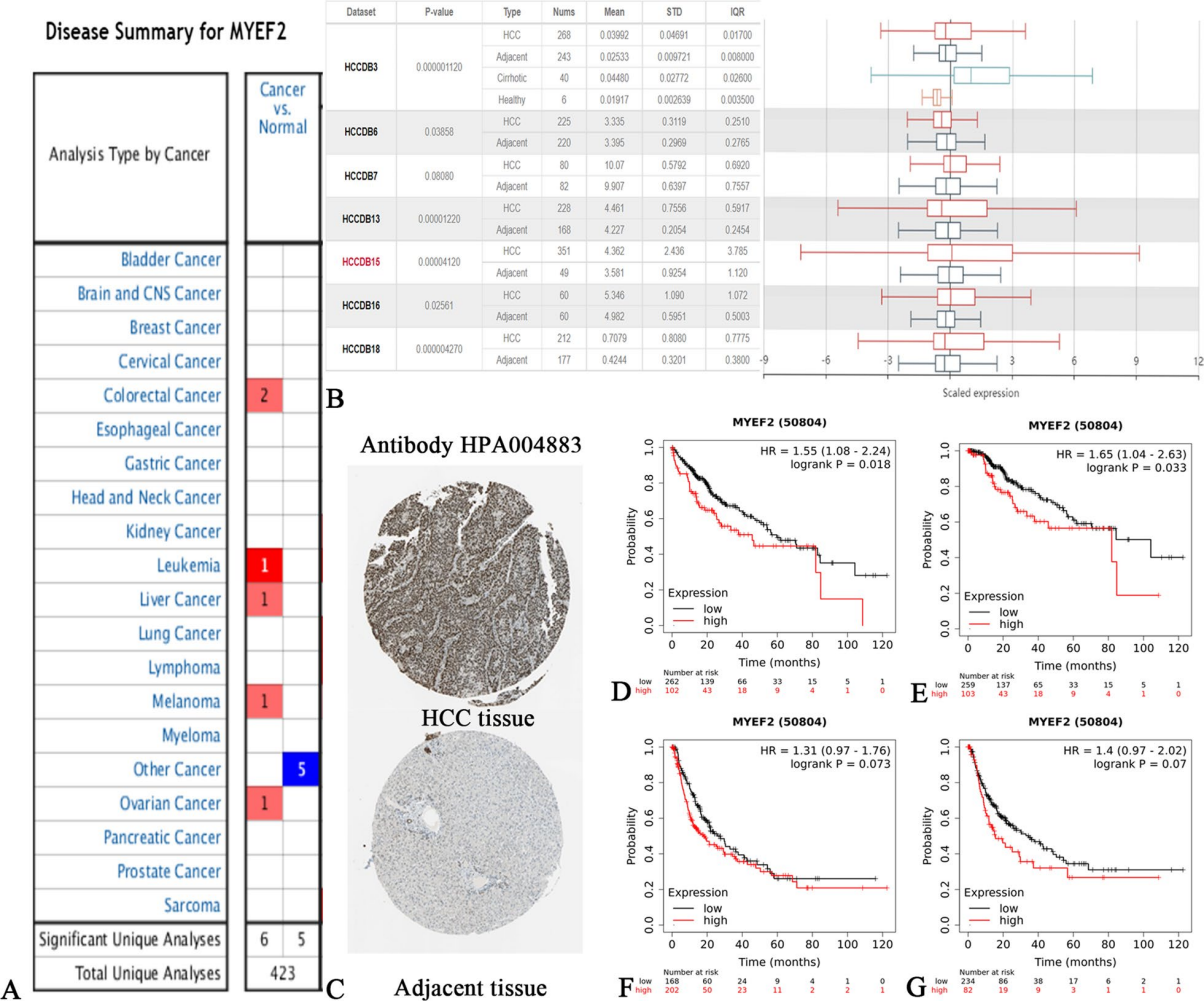
Preliminary screen of HCC biomarkers. **A**, **B** Dysregulated expression of MYEF2 in datasets containing different tumour tissues compared with normal tissues. **C** Expression of the MYEF2 protein in HCC from THE HUMAN PROTEIN ATLAS. **D** OS curve from the Kaplan–Meier Plotter database showing the prognostic difference between patients with high expression of MYEF2 and patients with low expression of MYEF2. **E** DSS curve from the Kaplan–Meier Plotter database showing the difference in the prognosis between patients with high MYEF2 expression and patients with low MYEF2 expression. **F** PFS curve from the Kaplan–Meier Plotter database showing the difference in the prognosis between patients with high MYEF2 expression and patients with low MYEF2 expression. **G** The RFS curve from the Kaplan–Meier Plotter database showed differences in the prognosis between patients with high MYEF2 expression and patients with low MYEF2 expression. MYEF2, myelin expression factor 2

We screened 423 studies to assess MYEF2 expression in different tumours from the ONCOMINE database. The results of 11 studies were statistically significant. Six studies reported high MYEF2 expression and 5 studies reported low MYEF2 expression. MYEF2 expression was upregulated in colorectal cancer, leukaemia, liver cancer, melanoma and ovarian cancer (Fig. 1A). Subsequently, we evaluated the MYEF2 expression level in several studies from the HCCDB database. The expression of MYEF2 in HCC tissues was generally upregulated compared with that in normal tissues (Fig. 1B). We obtained the expression of MYEF2 protein in 12 HCC tissues from THE HUMAN PROTEIN ATLAS, 8 cases of HCC tissues showed high expression, 2 cases showed moderate expression, 1 case showed low expression, and 1 case showed negative expression. MYEF2 was mainly expressed in the nucleus (Fig. 1C).

We searched the Kaplan–Meier Plotter database by setting conditions and identified 364 cases of HCC. The overall survival (OS) and disease-specific survival (DSS) of patients with high MYEF2 expression were significantly shorter than those of patients with low MYEF2 expression (*P* = 0.018 and *P* = 0.033, respectively) (Fig. 1D, E). Additionally, the progression-free survival (PFS) and recurrence-free survival (RFS) of patients with high MYEF2 expression were shorter than those of patients with low MYEF2 expression, although the results were not statistically significant (Fig. 1F, G). Taken together, MYEF2 was prognostically relevant with HCC and was a promising biomarker for predicting survival.

### Analysis of data from the cancer genome atlas database

**Fig. 2:**
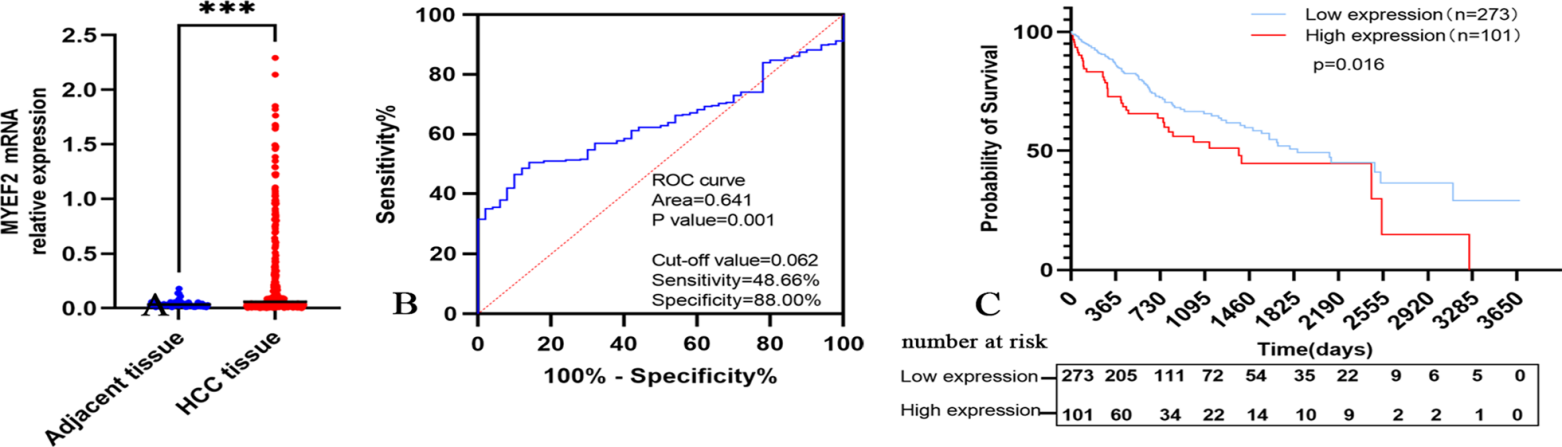
Analysis of data from The Cancer Genome Atlas database. **A** Images show the expression of the MYEF2 mRNA in HCC and adjacent tissues. **B** ROC curve for patients with HCC based on MYEF2 expression. **C** The prognostic value of MYEF2 expression in HCC. ***P < 0.001. MYEF2, myelin expression factor 2; ROC curve, receiver operating characteristic curve

We downloaded MYEF2 mRNA expression data from The Cancer Genome Atlas. The total sample number was 424, including 374 HCC tissue samples and 50 normal tissue samples. The database provided the basic characteristics of the patients. MYEF2 expression was upregulated in HCC (Fig. 2A). A receiver operating characteristic (ROC) curve was established to explore the diagnostic value of MYEF2. The outcome showed an AUC of 0.641 with a sensitivity of 48.66% and a specificity of 88.00%. The ideal cut-off value was 0.062 (Fig. 2B). We further analysed the correlations between MYEF2 expression and different pathological features (Table 1). MYEF2 expression in patients with advanced HCC (stage 2–4) was significantly higher than that in patients with early HCC (stage 1). Higher MYEF2 expression was detected in patients with high-grade (poorly differentiated) HCC (G3–G4) than in patients with low-grade (well differentiated) HCC (G1–G2). Significantly higher MYEF2 expression was observed in patients with T2–4 HCC than in patients with T1 HCC. The differences were statistically significant. In addition, the expression of MYEF2 in HCC tissues was not significantly correlated with age, lymph node metastasis, distant metastasis, alpha fetoprotein (AFP) level, total bilirubin (TB) level, albumin (Alb) level, creatinine (Cre) level, prothrombin time (PT) or platelet (Plt).

**Table 1:** Relationship between clinical features and MYEF2 expression in patients with liver cancer from The Cancer Genome Atlas database

Clinicopathologic features	MYEF2 expression		*P* value
	Low	High	
Gender			0.020
Male	194 (71.1%)	59 (58.4%)	
Female	79 (28.9%)	42 (41.6%)	
Age (years)	61 (52–69)	61 (51–69)	0.784
Tumor stage			0.004
I	138 (54.1%)	35 (36.8%)	
II–IV	117 (45.9%)	60 (63.2%)	
Degree of differentiation			0.001
G1–G2	183 (68.0%)	50 (50.0%)	
G3–G4	86 (32.0%)	50 (50.0%)	
Pathologic T^a^			0.006
T1	145 (53.7%)	38 (37.6%)	
T2–T4	125 (46.3%)	63 (62.4%)	
Distant metastasis			0.146
No	190 (69.6%)	78 (77.2%)	
Yes	83 (30.4%)	23 (22.8%)	
AFP (ug/L)	12 (4.00–144.25)	24 (3.75–1928.50)	0.206
TB (mg/dl)	1.2 (1.10–1.30)	1.2 (1.20–1.30)	0.277
Alb (g/dl)	4.0 (3.50–4.30)	4.1 (3.48–4.33)	0.662
Cre (mg/dl)	0.9 (0.70–1.10)	1.0 (0.80–1.10)	0.260
PT (s)	1.1 (1.00–9.45)	1.1 (0.95–9.00)	0.148
PLT (10^9^/L)	209 (158.00–303.00)	213 (162.75–285.50)	0.971
Vascular invasion			0.063
No	160 (68.4%)	48 (57.1%)	
Yes	74 (31.6%)	36 (42.9%)	

^a^According to TNM Classification by the American Joint Committee on Cancer (AJCC)

MYEF2, myelin expression factor 2; AFP, alpha fetoprotein; TB, total bilirubin; Alb, albumin; Cre, creatinine; PT, prothrombin time; PLT, platelet. Data are presented as nos. (%) or medians (interquartile ranges)

*P* values ≤ 0.05 indicate statistical significance

The survival analysis showed that the average survival time of 273 patients with low MYEF2 expression was 2015.97 ± 129.66 days and that of 101 patients with high MYEF2 expression was 1516.36 ± 174.68 days. MYEF2 expression was associated with the survival of patients with HCC. The prognosis of patients with HCC presenting high MYEF2 expression was significantly poor (Fig. 2C). A univariate analysis of each clinicopathological parameter was performed to examine its correlation with the survival of patients with HCC and identify variables with potential prognostic significance. The MYEF2 expression level, tumour stage, T stage, lymph node metastasis and distant metastasis were risk factors affecting the prognosis of patients with HCC. The multivariate analysis showed that MYEF2 expression and distant metastasis were independent risk factors for the prognosis of patients with HCC (Table 2).

**Table 2:** Univariate and multivariate Cox proportional hazard analyses of factors from The Cancer Genome Atlas database associated with overall survival

Factor	Univariable			Multivariable		
	Hazard ratio	95% confidence interval	*P*-value	Hazard ratio	95% confidence interval	*P*-value
MYEF2	0.636	0.439–0.920	0.016	0.670	0.451–0.995	0.047
Age (years)	1.190	0.840–1.687	0.328			
Gender	1.248	0.876–1.777	0.219			
Tumor stage	0.487	0.335–0.710	< 0.001	0.326	0.041–2.600	0.290
Tumor grade	0.898	0.628–1.258	0.557			
Pathologic T	0.479	0.334–0.685	< 0.001	1.563	0.201–12.121	0.669
Lymph node metastasis	0.676	0.469–0.973	0.035	1.122	0.662–1.903	0.669
Distant metastasis	0.613	0.426–0.883	0.009	0.567	0.337–0.954	0.032
AFP	0.910	0.572–1.450	0.692			

Univariate and multivariate analyses, Cox proportional hazards regression model

*OS* overall survival, *MYEF2* myelin expression factor 2, *AFP* alpha fetoprotein

*P* values ≤ 0.05 indicate statistical significance

### Increased MYEF2 expression in patients with HCC and cell lines

**Fig. 3:**
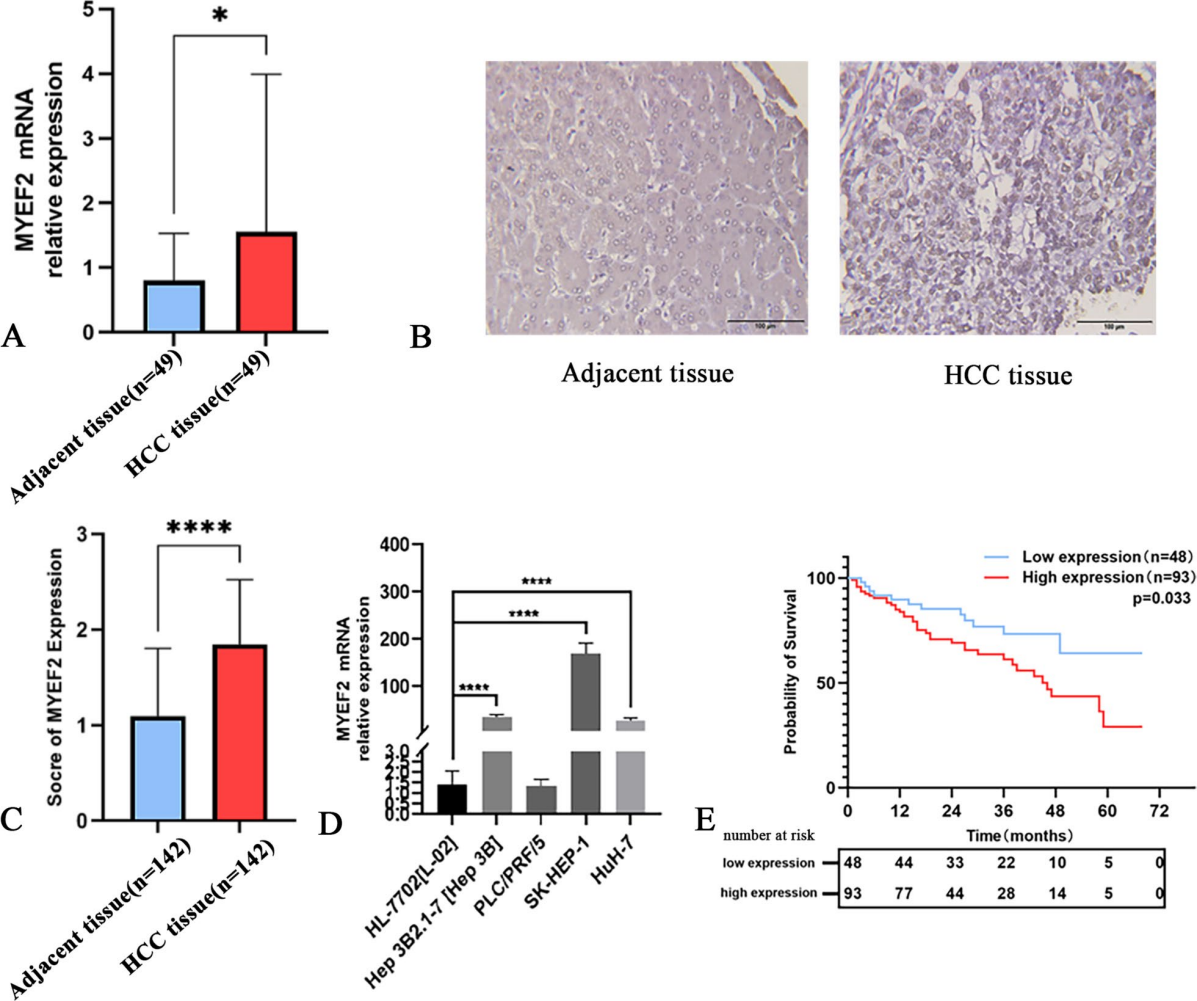
MYEF2 expression in HCC tissue and *cell lines*. **A** Fluorescent quantitative polymerase chain reaction was performed to detect MYEF2 mRNA expression in HCC (n = 49) and adjacent tissues (n = 79). **B** Representative images of immunohistochemical staining for MYEF2 in HCC and adjacent tissues. **C** Immunohistochemical score for MYEF2 expression in HCC and adjacent tissues. **D** Detection of MYEF2 mRNA expression using fluorescent quantitative polymerase chain reaction in HCC cell lines, including Hep 3B2.1-7, SK-HEP-1, HuH-7, and PLC/PRF/5, and in normal human liver HL-7702 (L-02) cells, which served as the control. **E** Kaplan–Meier plots of the overall survival of 276 patients with HCC stratified by MYEF2 expression. *P < 0.05, **P < 0.01, and ****P < 0.0001

We examined MYEF2 expression in HCC and adjacent normal liver tissues using qPCR. The expression of the MYEF2 mRNA was upregulated in HCC compared with adjacent normal liver tissues (*P* < 0.05, Fig. 3A). In addition, immunohistochemical staining was performed to detect the expression and cellular localization of MYEF2 in HCC and adjacent normal liver tissues. MYEF2 was mainly expressed in the nucleus (Fig. 3B). In 142 pairs of HCC tissues and adjacent normal tissues, the expression of the MYEF2 protein in HCC tissues was significantly higher than that in adjacent normal liver tissues (*P* < 0.05, Fig. 3C). Subsequently, we detected the expression of MYEF2 in HCC cell lines and normal liver cell lines. qPCR showed that compared with normal liver cell lines, the MYEF2 mRNA was expressed at high levels in HCC cell lines (Fig. 3D).

### Correlation of MYEF2 expression with patient survival

According to the difference between the HCC tissue staining score and adjacent normal tissue staining score, all patients were divided into two groups. A difference > 0 was considered the high expression group, and a difference ≤ 0 was considered the low expression group. All patients were followed for 3–5 years (Table 3). The Kaplan–Meier curve showed that relatively high MYEF2 expression was an important prognostic factor indicating shorter overall survival of patients. The survival rate of 93 patients with high expression was significantly lower than that of 48 patients with low expression (*P* = 0.033, Fig. 3E).

**Table 3:** Association between MYEF2 expression and pathological parameters of patients

Pathological parameter	MYEF2 expression		*P*-value
	High	Low	
All cases	93	48	
Gender			0.964
Male	72 (77.8%)	37 (78.1%)	
Female	21 (22.2%)	11 (21.8%)	
Age (years)			0.423
≤ 56	55 (58.7%)	25 (53.1%)	
> 56	38 (41.3)	23 (46.9%)	
P53 expression			0.224
Low expression	31 (33.3%)	21 (43.8%)	
High expression	62 (66.7%)	27 (56.2%)	
CK19 expression			0.614
Low expression	64 (68.3%)	35 (71.9%)	
High expression	29 (31.7%)	13 (28.1%)	
Hepatocyte expression			0.110
Low expression	50 (54.0%)	19 (40.6%)	
High expression	43 (46.0%)	29 (59.4%)	
Gly-3 expression			**0.004**
Low expression	29 (31.7%)	27 (56.2%)	
High expression	64 (68.3%)	21 (43.8%)	
Arg-1 expression			0.278
Low expression	61 (65.1%)	27 (56.2%)	
High expression	32 (34.9%)	21 (43.8%)	
AFP expression			**0.029**
Low expression	29 (31.7%)	24 (50.0%)	
High expression	64 (68.3%)	24 (50.0%)	
Vascular invasion			0.725
Negative	59 (64.3%)	29 (60.4%)	
Positive	34 (35.7%)	19 (39.6%)	

*MYEF2* myelin expression factor 2, *P53* tumour protein 53, *CK19* cytokeratin-19, *Arg-1* arginase 1, *AFP* alpha fetoprotein

*P* values ≤ 0.05 indicate statistical significance

Subsequently, we performed a univariate analysis on some pathological parameters to examine their correlations with the survival of patients. The hazard ratio and *P* values for each parameter were used to predict the prognosis of patients with HCC. The importance of each parameter was calculated by performing multivariate Cox proportional hazard analysis. The univariate analysis showed that MYEF2 expression and vascular invasion were important prognostic factors for patients with HCC. The multivariate analysis produced the same results as the univariate analysis (Table 4). Thus, MYEF2 expression was an independent risk factor affecting the prognosis of patients with HCC.

**Table 4:** Univariable and multivariable Cox proportional hazard analyses of pathological parameters associated with overall survival

Factor	Univariable			Multivariable		
	Hazard ratio	95% confidence interval	*P*-value	Hazard ratio	95% confidence interval	*P*-value
MYEF2	1.995	1.041–3.824	0.037	5.587	1.254–24.888	0.024
Gender	1.870	0.841–4.161	0.079			
Age (years)	0.967	0.552–1.692	0.905			
P53	0.925	0.505–1.694	0.800			
CK19	0.910	0.496–1.668	0.760			
Hepatocyte	1.329	0.738–2.392	0.334			
Gly-3	0.991	0.559–1.759	0.976			
Arg-1	1.192	0.648–2.919	0.572			
AFP	0.612	0.318–1.178	0.142			
Vascular invasion	0.013	0.000–0.877	0.043	0.000	0.000–3.485 × 10^94^	0.915

Univariate and multivariate analyses, Cox proportional hazards regression model

*OS* overall survival, *MYEF2* myelin expression factor 2, *P53* tumour protein 53, *CK19* cytokeratin-19, *Arg-1* arginase 1, *EGFR* epidermal growth factor receptor, *AFP* alpha fetoprotein

P values ≤ 0.05 indicate statistical significance

### MYEF2 promotes the invasion and migration of HCC cells in vitro

**Fig. 4:**
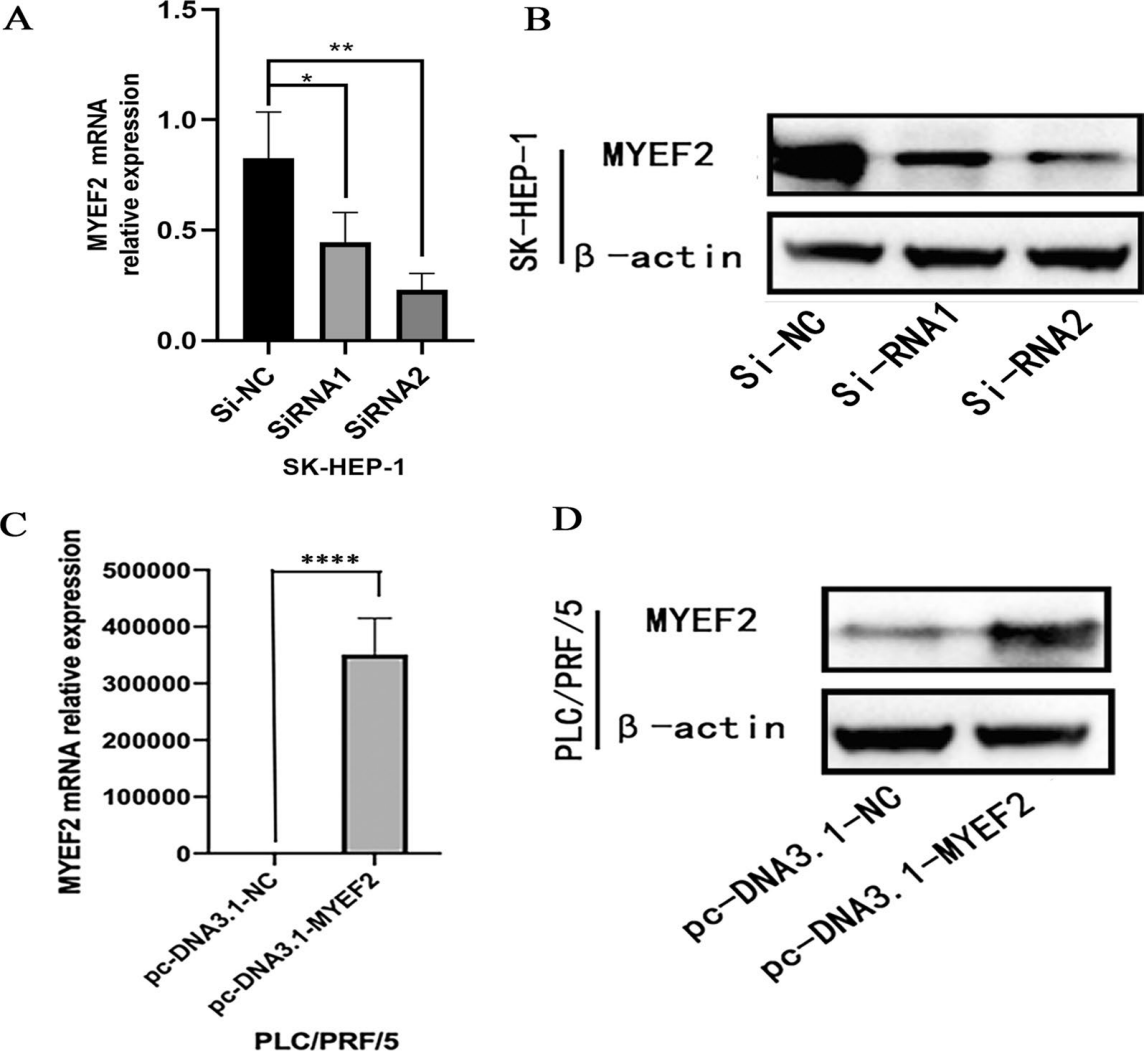
Validation of hepatocellular carcinoma cell model with MYEF2 knockdown and overexpression. **A**, **B** qPCR and Western blot were used to detect the knockdown efficiency of MYEF2. **C**, **D** qPCR and Western blot were used to detect the overexpression efficiency of MYEF2. *P < 0.05, **P < 0.01, and ***P < 0.001

We successfully established in vitro HCC cell models with MYEF2 knockdown and overexpression and verified the transfection efficiency by performing qPCR and western blotting (Fig. 4).

**Fig. 5:**
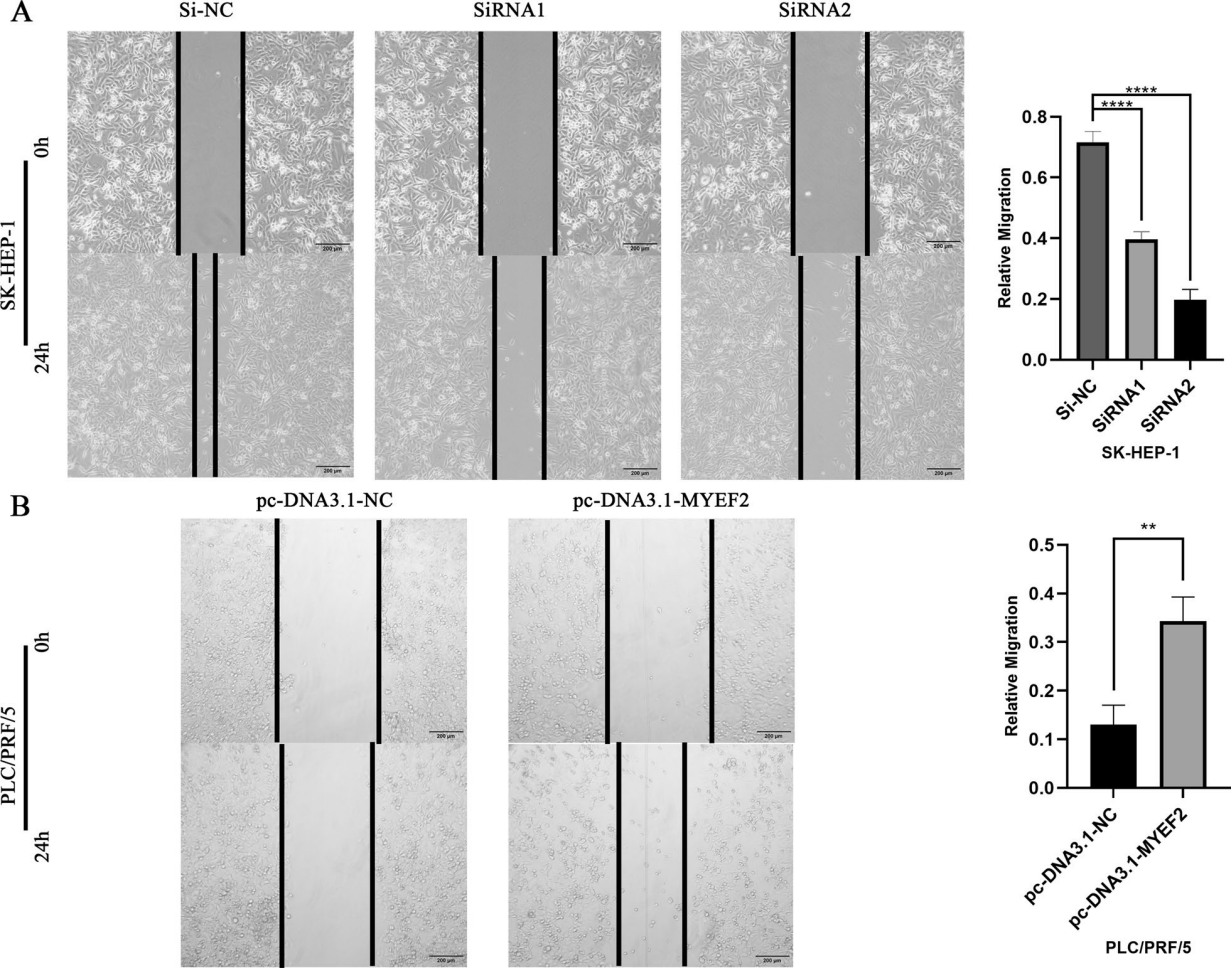
Effect of MYEF2 on migration of HCC cells by wound healing assay. **A** The effect of MYEF2 knock-down on SK-HEP-1 cell migration was analyzed by scratch test. **B** The scratch test analyzed the effect of MYEF2 overexpression on the migration ability of PLC/PRF/5 cells. **P < 0.01, and ****P < 0.0001

We examined the effect of MYEF2 on the migration ability of HCC cells through wound healing assay. The results showed that the scratch width of the experimental group (SiRNA1, SiRNA2) was significantly larger than that of the control group (Si-NC). After overexpression of MYEF2, compared with the control group (pc-DNA3.1-NC), the scratch width of the experimental group (pc-DNA3.1-MYEF2) was significantly reduced. Thus, MYEF2 might promote the migration of HCC cells (Fig. 5).

**Fig. 6:**
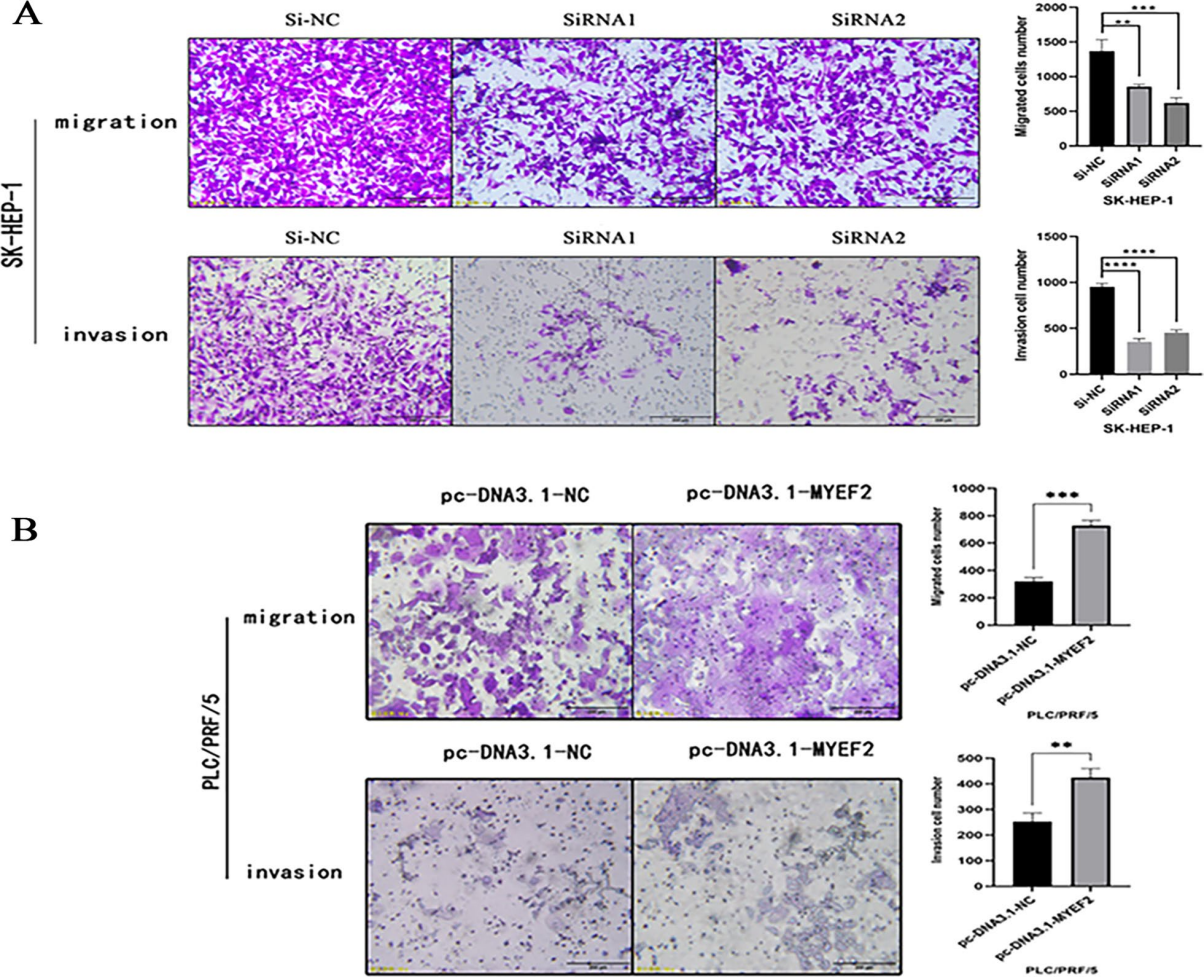
MYEF2 enhances invasion and migration of HCC cells. **A** Transwell assay was used to detect the effect of knock-down MYEF2 on the invasion and migration of SK-HEP-1 cells. **B** Transwell assay was used to detect the effect of overexpression of MYEF2 on the invasion and migration of PLC/PRF/5 cells. *P < 0.05, **P < 0.01, ***P < 0.001, and ****P < 0.0001

In addition, the Transwell assays showed that when MYEF2 was knocked down, cell invasion and migration were significantly reduced compared with the negative control group. In contrast, compared with the negative control group, MYEF2 overexpression increased invasion and migration of HCC cells (Fig. 6). In conclusion, MYEF2 may be involved in the development of HCC by enhancing the invasion and migration of HCC cells.

## Discussion

HCC is one of the leading causes of cancer-related mortality worldwide [2]. HCC has an occult onset and lacks specific clinical manifestations and typical symptoms in the early stage. Most patients with the middle and late stages of HCC and have lost the opportunity for effective treatment. Therefore, the 5-years survival rate of patients with HCC is less than 20% [22]. In addition, HCC has high recurrence and metastasis rates. Therefore, the identification of new tumour markers, early detection, early diagnosis and early treatment are key to improving the survival rate of patients with HCC [7].

Our team has investigated new tumour biomarkers for many years [23, 24]. However, most of our experimental results are based on dozens to hundreds of cases in our medical institution. The number of patients, ethnicity, geographical and other factors are limitations of our previous studies, and reliable and effective biomarkers have not been determined. The combination of bioinformatics methods and experimental studies may be good to overcome this shortcoming. With the wide application of gene-related technologies such as gene chips and NGS, a large number of core slice data have been generated, and most of the data have been stored in public databases [25, 26]. Therefore, integrating and reanalysing these datasets may provide valuable clues for new research [27]. In the present study, we first used a public database integrating a large number of samples to discover and identify a potential liver cancer marker, MYEF2, which improved the quality and credibility of the research to better perform subsequent experimental studies.

MYEF2 is a transcriptional repressor that mainly inhibits the transcription of the myelin basic protein gene (MBP) by binding to the proximal MB1 element 5′-TTGTCC-3′ of the MBP promoter, thereby participating in brain development [28]. In recent years, researchers have found that MYEF2 is mainly expressed in poorly differentiated and undifferentiated cells; for example, it plays a role in the formation of red blood cells by binding RUNX1. This effect gradually disappears during differentiation [29]. In one study, researchers found that some tumour cells expel extracellular proteins (such as H1.0 histones) to resist cell proliferation by producing extracellular vesicles. For example, extracellular vesicles released by melanoma cells also contain the H1.0 mRNA. Therefore, they searched for their corresponding mRNA-binding proteins by performing a series of experiments and finally found that the most common mRNA-binding protein was MYEF2 [30]. In addition, current studies have confirmed that MYEF2 is involved in the development of lung adenocarcinoma and schwannoma [31, 32]. However, no report had been documented the expression and function of MYEF2 in HCC.

In the bioinformatics analysis phase of this study, We preliminarily found that MYEF2 was abnormally expressed in various malignant tumors, and MYEF2 was significantly overexpressed in HCC tissues compared with normal liver tissues. The expression of MYEF2 was significantly associated with the prognosis of HCC patients, and the prognosis of patients with high expression of MYEF2 was significantly poor, suggesting that MYEF2 might be a prognostic biomarker for HCC. Therefore, based on RNAeq and clinical data from the Cancer Genome Atlas database, we verified the previous conclusions and further explored the correlation between MYEF2 expression level and clinical parameters of patients. We found that the expression of MYEF2 was higher in HCC patients with late tumor stage, higher malignancy and poorer differentiation. MYEF2 may be a potential biomarker for evaluating the severity of HCC. In addition, we confirmed that MYEF2 had a certain diagnostic value for HCC by drawing the ROC curve, but the efficacy was poor. It remains to be studied whether it can be used as an effective biomarker for the diagnosis of HCC. Survival analysis confirmed that the prognosis of HCC patients with high expression of MYEF2 was significantly poor. Further univariate and multivariate analysis showed that the high expression of MYEF2 was an independent risk factor affecting the prognosis of HCC patients.

In the basic experimental research stage, we confirmed that the expression of MYEF2 mRNA and protein was upregulated in HCC tissues and that the MYEF2 protein was mainly located in the nucleus of HCC cells. qPCR results also showed the upregulation of MYEF2 in various HCC cell lines compared with the levels in normal hepatocytes. Based on these results, we concluded that MYEF2 expression is increased in HCC tissues and cell lines and may be related to tumour pathology or the prognosis of patients with HCC. We confirmed the relationship between MYEF2 expression and the prognosis of patients by dividing 142 patients into two groups according to the immunohistochemical staining score of tissue sections. The Kaplan–Meier curve showed that the overall survival of patients with high MYEF2 expression was shorter than that of patients with low MYEF2 expression. This result confirmed the correlation between MYEF2 expression and the prognosis of patients. Therefore, we further evaluated the prognostic value of MYEF2. Univariate and multivariate analyses showed that MYEF2 was an important factor affecting the prognosis of patients with HCC and an independent prognostic biomarker for HCC. In vitro experiments showed that cell invasion and migration were inhibited when MYEF2 was knocked down. In contrast, MYEF2 overexpression increased cell invasion and migration.

However, the current research only preliminarily confirmed the abnormal expression of MYEF2 in HCC and performed functional experiments. Therefore, further studies mayt design to clarify the mechanism by which MYEF2 participates in the occurrence and development of HCC are needed in the future.

## Conclusions

In summary, MYEF2 expression is upregulated in HCC, and is expected to become a biomarker for determining the severity and prognosis of the disease. In addition, it is also involved in the invasion and migration of HCC, indicating that MYEF2 may play important roles in the occurrence and development of HCC.

## Supplementary Information

**Additional file1.** The original images of Western blot experiments.

## Data Availability

The datasets generated and analyzed during the current study are available from the corresponding author on reasonable request. The raw data could also be obtained from online databases including ONCOMINE (https://www.oncomine.org/resource/login.html) (Services terminated), HCCDB (http://lifeome.net/database/hccdb/search.html), THE HUMAN PROTEIN ATLAS (https://www.proteinatlas.org), Kaplan–Meier Plotter (http://kmplot.com/analysis) and The Cancer Genome Atlas database (https://portal.gdc.cancer.gov/genes/ENSG00000104177) without any restrictions.

## Abbreviations

HCC: Hepatocellular carcinoma
MYEF2: Myelin expression factor 2
qPCR: Fluorescent quantitative polymerase chain reaction
FBS: Foetal bovine serum
DAB: 3,3′-Diaminobenzidine
OS: Overall survival
DSS: Disease-specific survival
PFS: Progression-free survival
RFS: Recurrence-free survival
ROC: Receiver operating characteristic
AFP: Alpha fetoprotein
TB: Total bilirubin
Alb: Albumin
Cre: Creatinine
PT: Prothrombin time
PLT: Platelet
NGS: “Next-generation”, sequencing technology
