# Supplementary figures and images for: Expression and function of myelin expression factor 2 in hepatocellular carcinoma
Source: BMC Gastroenterol. 2023 Jan 19;23:20. doi: 10.1186/s12876-023-02644-3 (PMC9854206; doi:10.1186/s12876-023-02644-3)

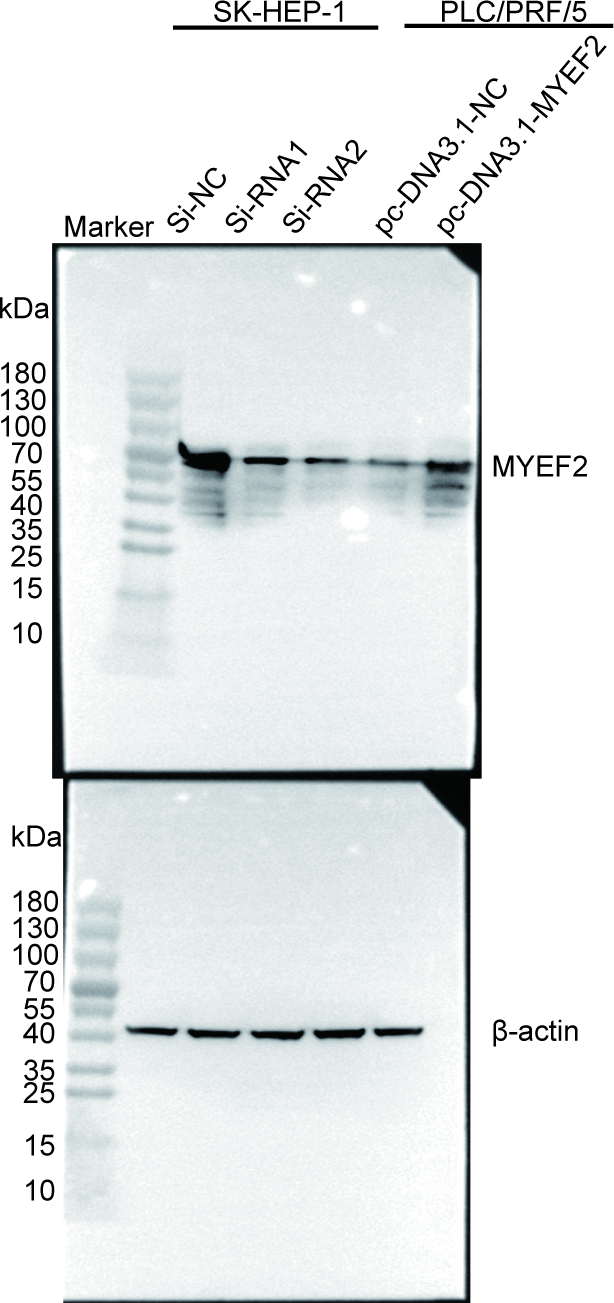

Supplement: Supplementary file 1 — Additional file1. The original images of Western blot experiments. [file 12876_2023_2644_MOESM1_ESM.tif]
